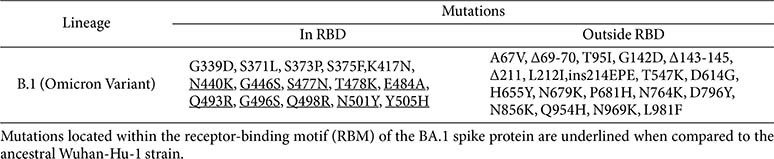# Domain-Specific Impacts of Spike Protein Mutations on Infectivity and Antibody Escape in SARS-CoV-2 Omicron BA.1

**DOI:** 10.4014/jmb.2507.07040

**Published:** 2025-09-16

**Authors:** Tae-Hun Kim, Sojung Bae, Jinjong Myoung

**Affiliations:** Korea Zoonosis Research Institute, Department of Bioactive Material Science and Genetic Engineering Research Institute, Jeonbuk National University, Jeonju 54531, Republic of Korea

**Keywords:** SARS-CoV-2, Omicron BA.1, domain, mutation, infectivity, immune evasion

## Abstract

The severe acute respiratory syndrome coronavirus 2 (SARS-CoV-2) epidemic began in Wuhan, China in late 2019, rapidly spreading worldwide and causing the COVID-19 pandemic. The virus evolved through multiple variants, with Omicron (first detected in late 2021) becoming dominant due to its extensive spike mutations, which enhanced immune evasion despite reduced infectivity compared to earlier strains. Here, we systematically evaluated the functional consequences of these mutations by generating pseudoviruses expressing spike proteins with domain-specific alterations. Mutations in the N-terminal domain (NTD) significantly enhanced pseudoviral infectivity, while receptor-binding domain (RBD) mutations markedly reduced infectivity. Importantly, NTD-mediated enhancement was attenuated when combined with RBD mutations, highlighting a complex interplay between spike regions. Despite lower infectivity compared to Delta, BA.1 pseudoviruses harboring RBD mutations exhibited robust resistance to neutralizing monoclonal antibodies, including casirivimab and imdevimab, with IC_50_ values exceeding assay limits. These findings indicate that Omicron BA.1’s rapid global spread is driven by enhanced immune evasion conferred by RBD mutations, even at the expense of viral entry efficiency. Our domain-specific analysis underscores the critical roles of spike protein mutations in shaping Omicron BA.1’s transmissibility and antibody escape, informing strategies for therapeutic and vaccine development.

## Introduction

The SARS-CoV-2 pandemic, originating in Wuhan, China in late 2019, has dramatically impacted global health, scientific research, and daily life. The virus’s rapid spread led to the COVID-19 pandemic, with its ongoing evolution marked by the emergence of multiple variants that have shaped the trajectory of the global crisis [1]. SARS-CoV-2 belongs to the Coronaviridae family and is closely related to SARS-CoV and MERS-CoV. Its genome is a single-stranded RNA of about 30,000 nucleotides, encoding several structural proteins, most notably the spike (S) protein. The spike protein itself is a trimeric, heavily glycosylated structure, with each protomer comprising an S1 subunit (containing the NTD and RBD) and an S2 subunit (involved in membrane fusion). The S1 subunit mediates host receptor binding, while S2 facilitates fusion of the viral and cellular membranes, a critical step for viral entry [2]. The spike’s structure and function have been extensively studied, revealing its conformational flexibility and the importance of its glycan shield in immune evasion [3, 4]. Other structural proteins, such as envelope (E), membrane (M), and nucleocapsid (N), play critical roles in viral assembly and genome protection.

As SARS-CoV-2 spread globally, it accumulated mutations, leading to the emergence of variants of concern (VOCs) such as Alpha, Beta, Gamma, Delta, and Omicron [1, 5, 6]. These variants have exhibited increased transmissibility, altered virulence, and importantly, varying abilities to evade immune responses generated by prior infection or vaccination. The D614G mutation in the spike protein was among the first widespread mutations, associated with increased viral loads and transmissibility [7-9]. The Delta variant, notable for mutations such as T478K, L452R, and P681R, was marked by high infectivity and fusogenicity, contributing to large waves of infection worldwide [10-13].

The Omicron variant, first identified in late 2021, rapidly supplanted Delta as the dominant global strain [6, 14-18]. Omicron, particularly the BA.1 lineage, is distinguished by an unprecedented number of spike protein mutations-over 30 in the spike alone [19], with 15 in the RBD-many of which alter key functional domains involved in receptor binding and immune recognition. Notable mutations include N501Y, which increases ACE2 binding affinity and transmissibility; K417N and E484A, which contribute to immune escape by altering antibody recognition sites; and P681H, associated with increased cleavage efficiency at the furin site, further enhancing infectivity [6]. The D614G mutation, present in earlier variants, also remains in Omicron and contributes to viral fitness [8, 9]. These spike mutations have profound effects on the virus’s biology. Omicron BA.1 is less dependent on the host protease TMPRSS2 [8] and is less efficient in spike cleavage and cell-cell fusion compared to earlier variants, which may affect tissue tropism and pathogenicity [14, 20]. The variant’s altered entry pathway, favoring endosomal over plasma membrane fusion, and reduced fusogenicity have been linked to specific spike mutations, such as H655Y, S375F, and the 69-70 deletion. These changes contribute to Omicron’s distinctive virological features and may underlie its reduced virulence compared to Delta, despite its high transmissibility [6].

A defining feature of Omicron BA.1 is its remarkable ability to evade neutralizing antibodies [15]. The extensive set of spike mutations, particularly in the RBD and NTD, enables Omicron to escape from antibodies elicited by prior infection or vaccination [16, 21] to an extent not seen with previous variants [22, 23]. This immune evasion has led to increased rates of reinfection and breakthrough infections, even among fully vaccinated individuals, and has necessitated the development and deployment of updated vaccines and booster doses tailored to circulating variants.

Efforts have been made to delineate the roles of mutations encoded in spike proteins of Omicron BA.1 especially in terms of infectivity or immune escape. Most of mutational studies on the spike protein of Omicron BA.1 were focused on the receptor binding domain, which is important for both receptor interactions and neutralization by antibodies. In addition, those studies were performed mostly using pseudoviruses encoding each individual mutation. Therefore, it is hard to understand the independent or concerted effects of several to many mutations found in the spike protein. Moreover, it would be interesting to test if domains, other than the RBD, are analyzed for their unique contributions to viral transmissibility or antibody evasion. In this study, we generated a series of pseudoviruses harboring mutations grouped together as NTD, RBD, receptor binding motif (RBM), or C terminal domain (CTD) in the presence of D614G mutation. And thus, unique contributions of each domain containing many different mutations were analyzed as well as those of combined domains. Interestingly, our study demonstrated that domain-specific roles in viral transmissibility or antibody evasion were evident. These studies will provide an important rationale for the development of antiviral countermeasures including vaccines and therapeutics.

## Materials and Methods

### Cells and Reagents

HEK 293T cells (ATCC, CRL-3216) were cultured in Dulbecco’s Modified Eagle Medium (DMEM; Welgene, Republic of Korea) containing high glucose, supplemented with 10% fetal bovine serum (FBS; Welgene) and 1%penicillin-streptomycin (P/S; Welgene, Republic of Korea) [17, 18, 24, 25]. HCC15-ACE2 cells, a stable HCC15 lung carcinoma cell line (ATCC, CCL-225) expressing the human angiotensin-converting enzyme 2 (hACE2) receptor via lentiviral transduction, were maintained in Roswell Park Memorial Institute 1640 medium (RPMI 1640; Welgene) supplemented with 10% FBS and 1% P/S. All cell lines were incubated at 37°C in a humidified atmosphere containing 5% CO_2_

### Mutagenesis of the Spike Protein of the SARS-CoV-2 Wuhan-Hu-1 Strain

The SARS-CoV-2 spike gene was codon-optimized for mammalian expression by GeneArt (Thermo Fisher Scientific, USA) and subcloned into the pcDNA3.1 vector (Invitrogen, USA) as previously described. Site-directed mutagenesis to introduce one or more mutations into the spike gene was performed using inverse sequence and ligation-independent cloning (SLIC) with specifically designed primers, in which the mutated codons were indicated. A67V_Δ69-70 forward primer; 5'-AACTACCGGTACCGGCTGTTCCGGAAG-3', A67V_Δ69-70 reverse primer; 5'-CCGGTACCGGTAGTTGTAGTTGCCGCCG-3', T95I forward primer; 5'-GCAGCAAGCCTTGTAACGGCGTGGAAGG-3', T95I reverse primer; 5'-TACAAGGCTTGCTGCCGGCCTGATAG-3', G142D_Δ143-145 forward primer; 5'-GGCGTGCAGGGCTTCAACTGCTACTTCCCAC-3', G142D_Δ143-145 reverse primer; 5'-GAAGCCCTGCACGCCGTTACAAGGGGTG-3', Δ211_L212I_ins214EPE forward primer; 5'-TATCAGGGCGTGAACTGTACAGAGGTGCCCGTGG-3', Δ211_L212I_ins214EPE reverse primer; 5'-GTTCACGCCCTGATACAGCACGGCCACCTGATTG-3', G339D forward primer; 5'-AATAGCCGGAGA CGGGCCAGAAGCG-3', G339D reverse primer; 5'-CCGTCTCCGGCTATTTGTCTGTGTCTGGTAGC-3', S371L_S373P_S375F foward primer; 5’-CTGGCCCCCTTCTTCACCTTCAAGTGCTACGGCG-3’, S371L_S373P_S375F reverse primer; 5’-GAAGAAGGGGGCCAGATTGTACAGCACGCTGTAGTCG-3’, K417N forward primer; 5'-CAGGCAACATCGCCGATTACAACTACAAGCTGCCCGAC-3', K417N reverse primer; 5'-CGGCGATGTTGCCTGTCTGTCCAGGAGCGATCTGC-3', G446S forward primer; 5’-CTGGACTCCAAAGTCAGCGGC AACTACAACTAC-3’, G446S reverse primer; 5’-GACTTTGGAGTCCAGCTTGTTGCTATTCCAGG-3’, S477N_T478K forward primer; S477N_T478K reverse primer; 5’-GGCAACAAGCCTTGTAACGGCGTGGAAG-3’, 5’-ACAAGGCTTGTTGCCGGCCTGATAGATTTCG-3’, E484A forward primer; 5’-GGCGTGGCCGGCTTC AACTGCTACTTCCCAC-3’, E484A reverse primer; 5’-GAAGCCGGCCACGCCGTTACAAGGGG-3’, Q493R_G496S_Q498R_N501Y forward primer; 5’-GTCCTACAGCTTTCGGCCTACCTACGGCGTGG-3’, Q493R_G496S_Q498R_N501Y reverse primer; 5’-CGAAAGCTGTAGGACCGCAGTGGGAAGTAGCAGTTGAAG-3’, Y505H forward primer; 5’-GTGGGCCACCAGCCCTATAGAGTGGTGGTG-3’, Y505H reverse primer; 5’-GGGCTGGTGGCCCACGCCGTAGGTAG-3’, T547K forward primer; 5’-GGCCTGAAGGGCACAGGCGTGCTGAC-3’, T547K reverse primer; 5’-TGTGCCCTTCAGGCCGTTGAAGTTGAAGTTC-3’, D614G forward primer; 5'-TATCAGGGCGTGAACTGTACAGAGGTGCCCGTGG-3', D614G reverse primer; 5'-GTTCACGCCCTGATA CAGCACGGCCACCTGATTG-3', H655Y forward primer; 5’-AGCCGAGTACGTGAACAATAGCTACGAGTGC-3’, H655Y reverse primer; 5’-TTCACGTACTCGGCTCCGATCAGACATCC-3’. Expression of the spike protein variants was assessed by Western blot analysis using polyclonal anti-spike S2 antibodies (Sino Biological, China).

### Generation of Lentivirus Pseudo-Typed with SARS-CoV-2 Spike Protein

Pseudo-typed lentiviruses expressing the SARS-CoV-2 spike protein, either of Wuhan-Hu-1 or containing mutations characteristic of the Omicron variant (BA.1 lineage), were generated by co-transfecting HEK293T cells with plasmids encoding HIV-1 gag-pol (psPAX2), the SARS-CoV-2 spike protein (with or without mutations), and a lentiviral vector backbone (pHR encoding firefly luciferase), as previously described. DNA transfection was performed using the TransIT-Lenti Transfection Reagent (Mirus Bio LLC, USA) according to the manufacturer’s protocol [17, 18, 26]. Seventy-two hours post-transfection, culture supernatants containing pseudoviruses were collected and clarified by centrifugation at 3,600 rpm for 5 min at 4°C to remove cellular debris. The pseudoviruses were aliquoted and stored at -80°C until further use. Viral titers were determined by quantitative reverse transcription PCR (qRT-PCR). Briefly, pseudoviral RNA was extracted and purified using the QIAamp Viral RNA Mini Kit (Qiagen, Germany), followed by single-step qRT-PCR using the Lenti-X qRT-PCR Titration Kit (Takara, Japan), in accordance with the manufacturer’s instructions [27].

### Measurement of Pseudovirus Infectivity

Pseudoviruses were titrated and normalized to ensure equivalent numbers of viral particles were used in subsequent infectivity assays. HCC15-ACE2 stable cells, which exhibit high susceptibility to SARS-CoV-2 infection (unpublished data), were infected with the normalized pseudoviruses. At 72 h post-infection, cells were lysed on ice for 5 min using chilled Reporter Lysis 5X Buffer (Promega, USA), followed by centrifugation at 15,000 rpm for 15 min at 4°C to remove cellular debris. Subsequently, 25 μl of each lysate was transferred to a new tube and mixed with 25 μl of Luciferase Assay buffer containing the luciferase substrate (Luciferase Assay System, Promega) for downstream analysis [28].

### SARS-CoV-2 Omicron Variant (BA.1 lineage) Virus

The SARS-CoV-2 Omicron variant (BA.1) was obtained from the Korea Center for Disease Control (KCDC, NCCP#43408) and propagated once in Vero-E6 cells prior to use at a multiplicity of infection (MOI) of 0.1. Culture supernatants containing virus were harvested 60 hours post-infection, and viral stocks were titrated using a plaque assay as previously described. All procedures involving live SARS-CoV-2 and its variants were conducted in a biosafety level 3 facility in accordance with the guidelines of the Institutional Biosafety Committee of Jeonbuk National University.

### Plaque Assay

Vero-E6 cells were seeded in 6-well plates and incubated overnight at 37°C in a humidified atmosphere with 5% CO_2_. Upon reaching approximately 90% confluency, viral stocks were serially diluted ten-fold in serum-free DMEM high glucose medium (Welgene). After serial dilution, the culture medium was replaced with the diluted viral samples, and the cells were incubated for 1 h at 37°C with 5% CO_2_ to allow viral adsorption. Subsequently, the cells were washed with Dulbecco’s Phosphate Buffered Saline (DPBS; Welgene) and overlaid with DMEM (Welgene) supplemented with 1% agarose, 2% fetal bovine serum (FBS; Gibco), and 1% penicillin-streptomycin (Gibco).

### Neutralization Assay

Pseudoviruses or live SARS-CoV-2 Omicron variant (BA.1) viruses were incubated with the indicated monoclonal antibodies, either individually or in combination, at 37°C for 1 h prior to infection of Vero-E6 cells. Following incubation, cells were thoroughly washed and subsequently incubated for 6 h (live SARS-CoV-2) or 72 h (pseudoviruses) before downstream analysis. Quantitative RT-PCR was performed for live virus-infected cells, while luciferase assays were conducted for pseudovirus-infected cells. The percentage of neutralization was calculated using the formula: (value(untreated) – value(treated)) × 100 / value(untreated) (%). Data were analyzed and plotted using GraphPad Prism 8.0, and nonlinear regression (curve fitting) was employed to determine the 50% inhibitory concentration (IC_50_).

### Quantitative Real-Time Reverse Transcription Polymerase Chain Reaction (qRT-PCR)

Viral RNA was extracted from 140 μl of virus-containing media using the GENTI Advanced Viral RNA Extract Kit in conjunction with the GENTI 32 Advanced Automatic Extraction Equipment (GeneAll, Republic of Korea), according to the manufacturer’s instructions. The extracted RNA was subsequently subjected to quantitative reverse transcription PCR (qRT-PCR) using the DiaStar OneStep Multiplex qRT-PCR Kit (SolGent, Republic of Korea) with the following primers and probe set: forward primer; 5’-GACCCCAAAATCAGCGAAAT-3’, reverse primer; 5’-TCTGGTTACTGCCAGTTGAA-3’, probe; 5’(FAM)-CGCATTACGTTTGGTGGACCCTCA-(TAMRA)3’.

### Statistical Analysis

Statistical analyses were conducted using Student’s *t*-test. A *p*-value of less than 0.05 was considered statistically significant. Statistical significance was denoted as follows: *p < 0.05; **p < 0.01; ***p < 0.001; ns, not significant (*p* ≥ 0.05). Data are presented as mean ± standard deviation (SD)

## Results

### Domain-Specific Mutational Analysis of the SARS-CoV-2 Omicron BA.1 Spike Reveals Enhanced Infectivity associated with NTD

**Fig. 1:**
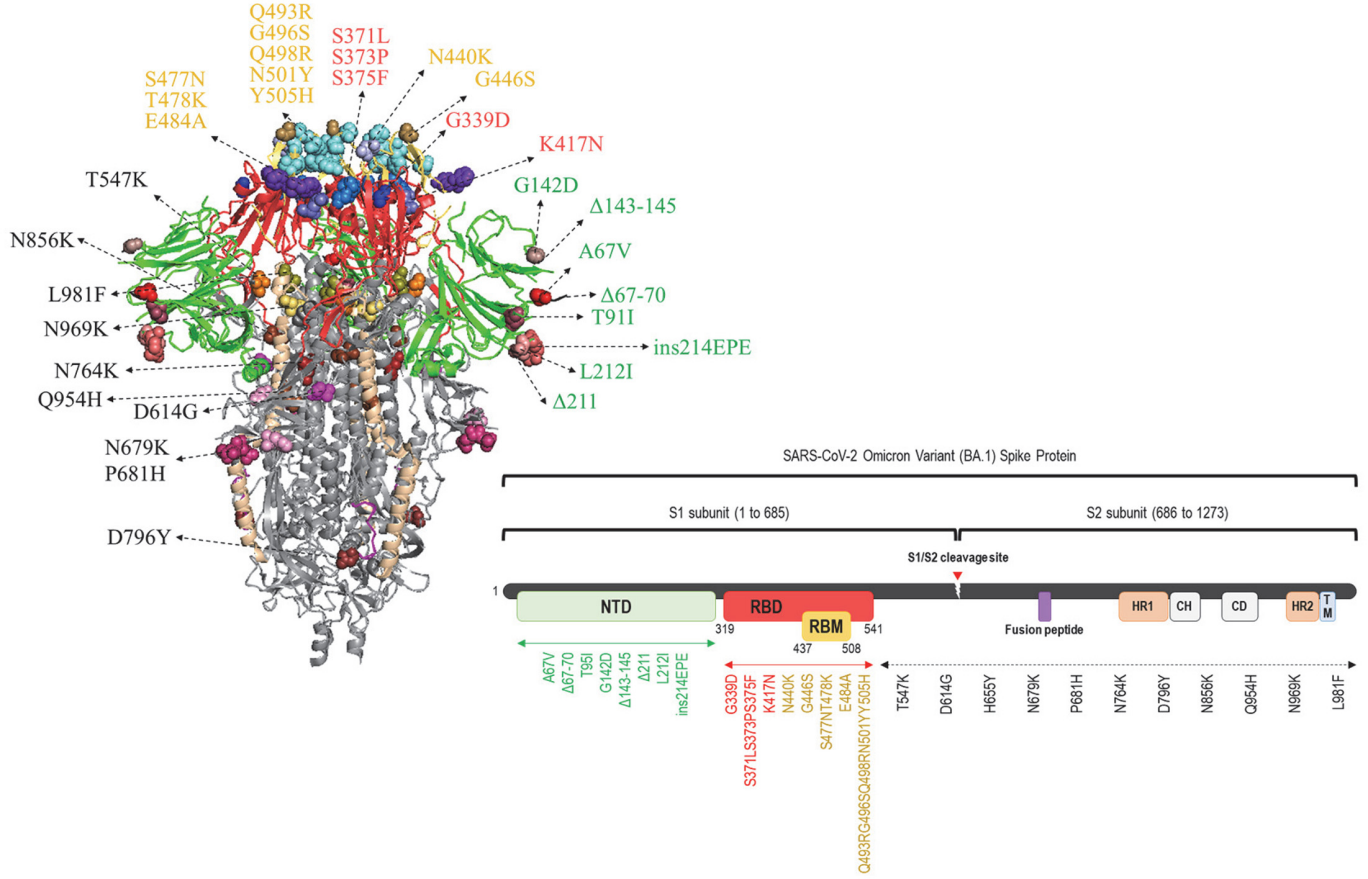
Schematic diagram of the SARS-CoV-2 BA.1 lineage spike protein. The illustration depicts the key mutations in the spike protein of SARS-CoV-2 BA.1 lineage.

**Fig. 2:**
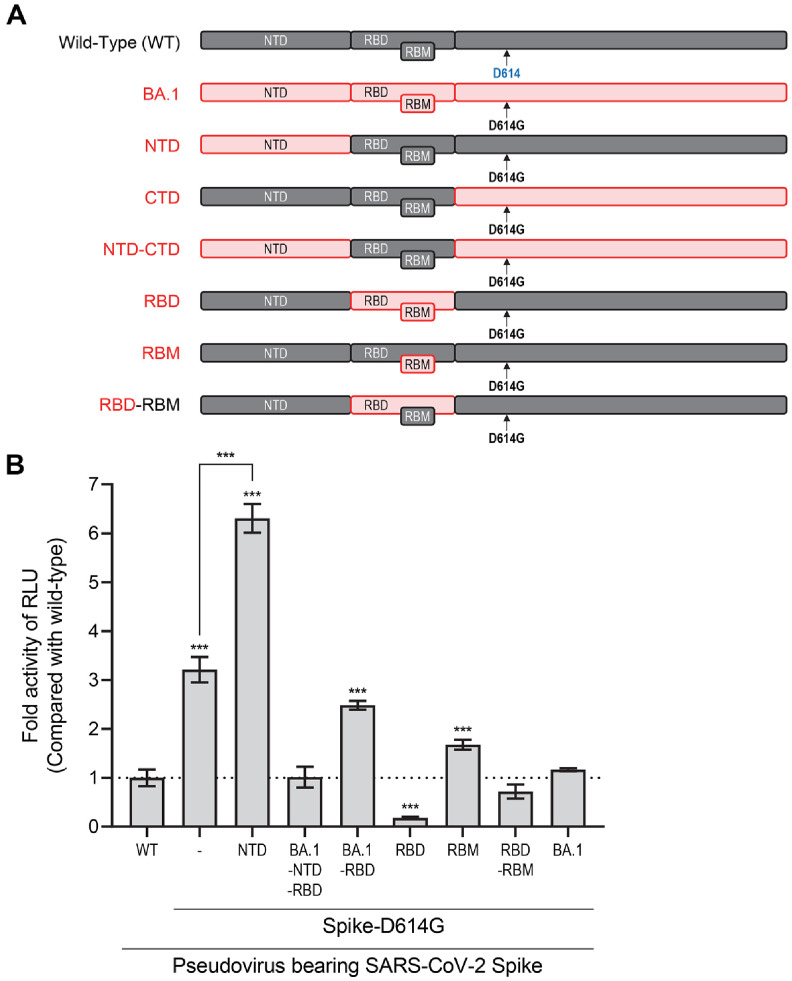
NTD mutations in the BA.1 spike protein significantly enhance viral infectivity. (**A**) Schematic diagram of spike protein constructs containing region-specific Omicron BA.1 mutations used to generate pseudotyped viruses. Mutations indicate amino acid changes relative to the ancestral Wuhan-Hu-1 strain. (**B**) ACE2-expressing HCC-15 cells were infected with pseudoviruses carrying either Wuhan-Hu-1 or variant spike proteins. Seventy-two hours after infection, cells were lysed and luciferase activity was measured. Relative luciferase units (RLU) were normalized to Wuhan-Hu-1, and fold changes are presented. Data represent three independent experiments, with error bars indicating mean ± SEM of technical replicates. Statistical significance was determined using Student’s *t*-test. *p < 0.05, **p < 0.02, ***p < 0.001.

The SARS-CoV-2 Omicron variant (BA.1) is characterized by an extensive array of mutations, particularly concentrated in the spike (S) protein, which distinguishes this lineage from earlier variants. For a detailed overview of these mutations, refer to Fig. 1 and Table 1. To systematically evaluate the functional consequences of these mutations, we categorized them by spike domain or region and constructed a series of spike-expressing vectors accordingly (Fig. 1 and 2A).

To assess the impact of individual spike regions from the BA.1 lineage, we generated pseudoviruses by pseudotyping with each spike construct. These pseudoviruses were used to infect hACE2-expressing HCC15 cells [17, 18], a model highly susceptible to SARS-CoV-2 infection, enabling a comparative analysis of viral infectivity (Fig. 2B). Notably, although the NTD of the spike does not directly mediate hACE2 binding, mutations within the NTD significantly enhanced pseudoviral infectivity compared to pseudoviruses bearing the Wuhan-Hu-1 or D614G spike. This finding highlights the potential contribution of NTD mutations to the increased transmissibility and altered phenotypic properties observed in the Omicron BA.1 variant.

### Distinct Functional Effects of NTD and RBD Mutations in the Omicron BA.1 Spike on Pseudoviral Infectivity

Mutations within the NTD of the Omicron BA.1 spike protein markedly enhanced pseudoviral infectivity. In contrast, mutations introduced into other spike domains or regions did not confer a similar increase in infectivity. Notably, pseudoviruses harboring

RBD mutations exhibited a significant reduction in infectivity compared to those expressing the Wuhan-Hu-1 spike, demonstrating an opposing effect to that observed with NTD mutations. Furthermore, the infectivity-enhancing effect of NTD mutations was diminished when combined with mutations in other spike regions. Specifically, co-expression of RBD mutations alongside NTD mutations resulted in a significant decrease in infectivity relative to pseudoviruses bearing NTD mutations alone (Fig. 2B). Collectively, these findings indicate that while NTD mutations contribute to increased infectivity of the BA.1 lineage, RBD mutations substantially impair infectivity, highlighting the complex interplay of spike domain mutations in shaping Omicron BA.1 viral entry characteristics.

### Omicron BA.1 RBD Mutations in the Spike Protein Confer Potent Neutralizing Antibody Escape although They May Diminish Viral Fitness

Although the Delta variant demonstrated high infectivity [17], attributed to the T478K and L452R mutations in the spike protein and increased fusogenicity via the P681R mutation, the Omicron BA.1 lineage rapidly supplanted Delta as the predominant variant by December 2021. The predominance of the BA.1 subvariant over the Delta variant is attributable to its substantial capacity to evade neutralizing antibodies, thereby promoting extensive transmission even within highly vaccinated populations. Additionally, increased infectivity of BA.1 in the upper respiratory tract may confer further selective advantages relative to the Delta variant [29].

**Fig. 3:**
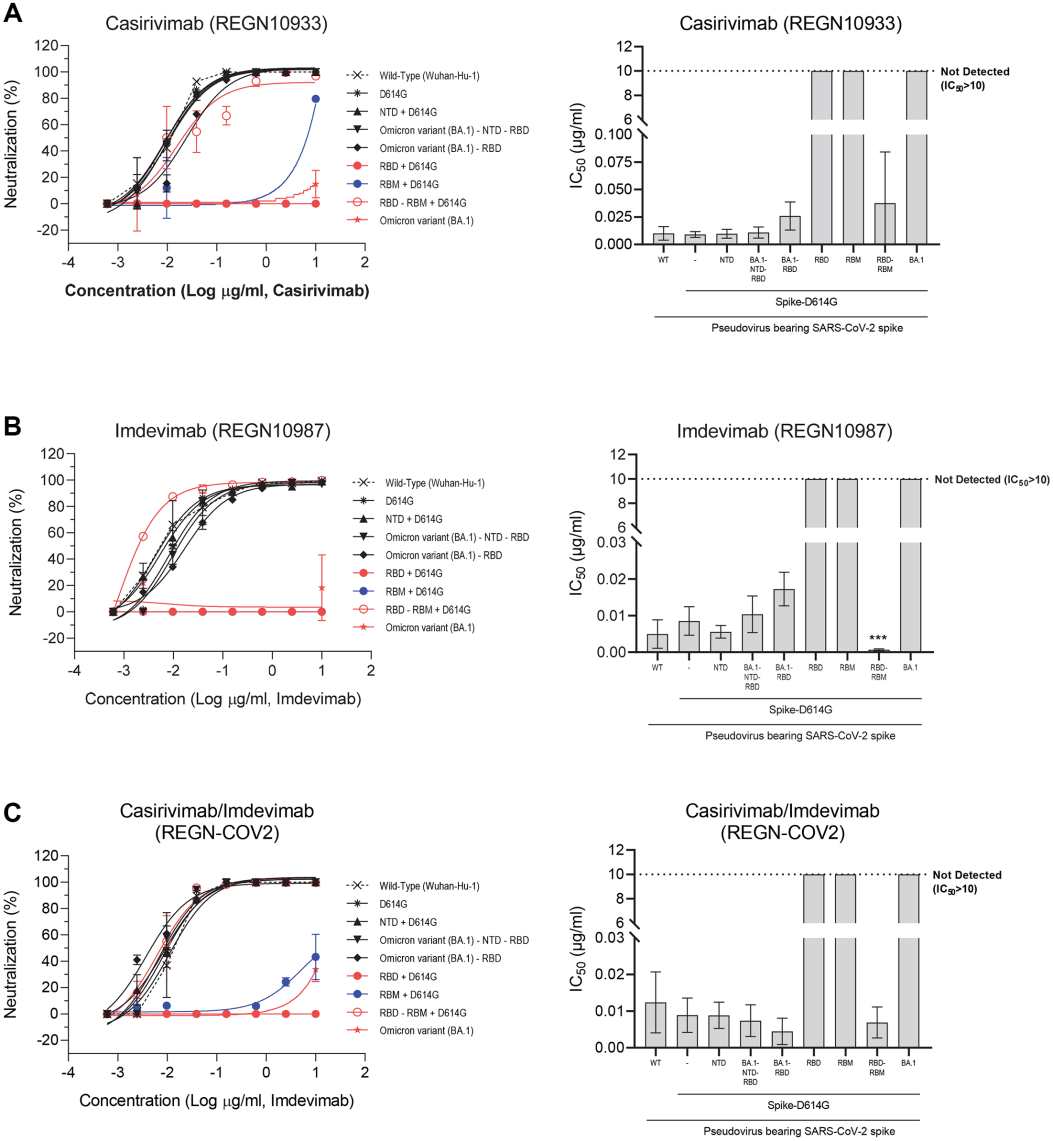
Marked antibody evasion mediated by RBD mutations in the BA.1 spike protein. Neutralization assays were performed using pseudoviruses bearing distinct spike protein mutations. Pseudoviruses were pre-incubated for 1 h at 37°C with (**A**) Casirivimab, (**B**) Imdevimab, or (**C**) a combination of both antibodies prior to infection of ACE2-expressing target cells. Seventy-two hours post-infection, cells were lysed, and luciferase activity was determined. Left panels: Doseresponse neutralization curves with data points representing mean ± SEM of percent neutralization relative to untreated controls, from two independent experiments performed in triplicate. Right panels: Half-maximal inhibitory concentrations (IC_50_) for each antibody or antibody combination against the pseudovirus variants. Bars indicate mean IC_50_ values from two independent experiments; IC_50_ calculations are detailed in the Materials and Methods. Individual figure legends for panels A, B, and C are presented below each respective panel. Statistical significance was determined using Student’s *t*-test by comparing each variant to the Wuhan-Hu-1. *p < 0.05, **p < 0.01, ***p < 0.001.

To evaluate the immune evasion capacity of the BA.1 lineage and pinpoint specific spike mutations responsible, we conducted neutralization assays using pseudoviruses expressing various spike mutations (Fig. 3). Pseudoviruses were pre-incubated with monoclonal antibodies (Casirivimab and Imdevimab) at 37°C for one hour before infection, as detailed in Materials and Methods. Neutralization percentages and half-maximal inhibitory concentrations (IC_50_) were calculated and visualized (Fig. 3).

Our results revealed that pseudoviruses bearing mutations in the receptor-binding motif (RBM) or elsewhere within the RBD consistently exhibited IC_50_ values exceeding 10 μg/ml for both antibodies, indicating a high level of resistance beyond the assay’s detection limit. Specifically, introduction of RBM mutations markedly enhanced resistance to Casirivimab (IC_50_ >10 μg/ml), which targets the RBM within the RBD (Fig. 3A). In contrast, pseudoviruses with only non-RBM RBD mutations remained sensitive to Imdevimab (IC_50_ = 0.0007 μg/ml), which binds outside the RBM (Fig. 3B). However, addition of RBM mutations also conferred significant resistance to Imdevimab.

Collectively, these findings demonstrate that BA.1 RBD mutations, particularly within the RBM, are key drivers of resistance to neutralization by both Casirivimab and Imdevimab. This robust immune evasion likely contributed to the rapid global spread and predominance of the Omicron BA.1 lineage.

### Enhanced Resistance of SARS-CoV-2 Omicron BA.1 to Neutralizing Antibodies despite Reduced Infectivity

**Fig. 4:**
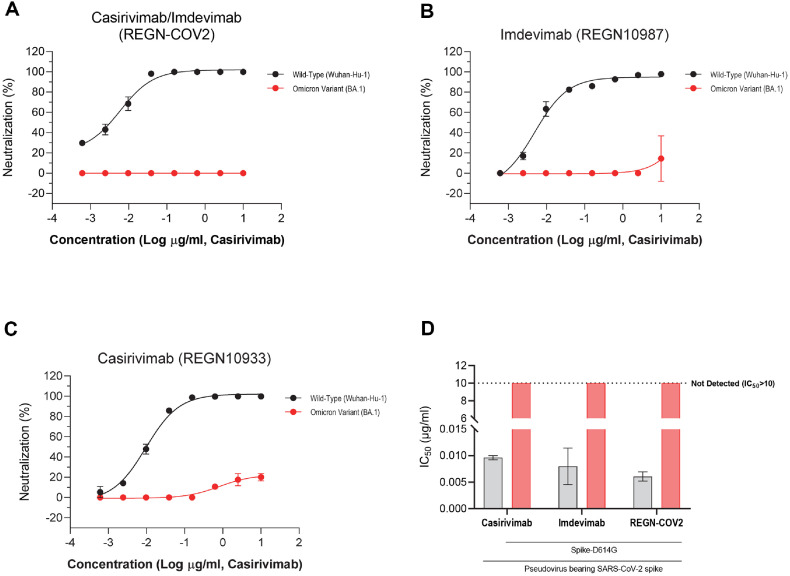
Complete neutralization escape of omicron variant by casirivimab and imdevimab compared to Wuhan-Hu-1. SARS-CoV-2 Wuhan-Hu-1 and Omicron (BA.1 lineage) viruses were pre-incubated with (**A**) Casirivimab, (**B**) Imdevimab, or (**C**) a combination of both antibodies at 37°C for 1 h. After incubation, virus–antibody mixtures were washed and used to infect target cells for 6 h, followed by quantification of intracellular viral RNA by qRT-PCR. Viral RNA levels were normalized to those of antibody-untreated controls. (**A–C**) Dose-response neutralization curves, with data points representing mean ± SEM percent neutralization from two independent experiments performed in triplicate. (**D**) IC_50_ for each antibody or antibody combination against the viral variants, shown as bars indicating mean IC_50_ values from two independent experiments. Detailed experimental protocols are provided in the Materials and Methods.

Building on our previous findings that pseudoviruses with RBD mutations display strong resistance to Casirivimab, Imdevimab, and their combination (REGN-COV2), we further evaluated the ability of the SARS-CoV-2 Omicron BA.1 lineage to evade these neutralizing antibodies. Wuhan-Hu-1 and the Omicron variant were pre-incubated with the indicated antibodies for one hour at 37°C before infecting VeroE6 cells. Intracellular viral titers were quantified by qRT-PCR, as described in the Materials and Methods. Neutralization percentages by Casirivimab and Imdevimab were plotted in Fig. 4A-4D. Consistent with expectations and recent reports [15, 16, 29], the BA.1 lineage exhibited markedly greater resistance to neutralizing antibodies compared to Wuhan-Hu-1. This enhanced immune evasion is attributed to the extensive set of mutations in the spike protein, particularly within the RBD, which disrupt antibody binding and diminish the efficacy of therapeutic monoclonal antibodies. Despite the reduced infectivity associated with RBD mutations in BA.1 (Fig. 2B), these mutations confer a significant advantage in antibody escape, as reflected in the substantially higher IC_50_ values observed for all tested antibodies (Figs. 3 and 4). Collectively, these results underscore the critical role of spike protein mutations in facilitating Omicron BA.1’s immune evasion, contributing to its rapid global spread despite lower intrinsic infectivity.

## Discussion

The emergence and global dominance of the SARS-CoV-2 Omicron variant (BA.1) marked a significant shift in the COVID-19 pandemic landscape. Unlike earlier variants such as Delta, which were characterized by high infectivity and fusogenicity, Omicron BA.1 rapidly supplanted Delta despite demonstrating lower intrinsic infectivity in vitro and attenuated pathogenicity in vivo. For example, In Calu-3 cells (human lung epithelial cell line), Omicron BA.1 showed 70% lower intracellular viral RNA compared to Delta at all time points [30] as well as live viral titers. Another study reported that BA.1 replication levels were more than 10-fold (1 log) lower compared to Delta [31]. In addition, the BA.1 varaint demonstrated dramatically reduced neurotropism as shown in neural cell model studies [32].

The present study provides a detailed domain-specific analysis of Omicron BA.1 spike protein mutations, elucidating their distinct contributions to viral infectivity and immune evasion. These findings offer critical insights into the molecular mechanisms underpinning Omicron’s epidemiological success and inform future strategies for vaccine and therapeutic design. Our results reveal that mutations within the NTD of the Omicron BA.1 spike protein significantly enhance pseudoviral infectivity in hACE2-expressing HCC15 cells (Fig. 2)[33, 34]. This observation is particularly notable given that the NTD does not directly mediate binding to the hACE2 receptor, which is the primary entry point for SARS-CoV-2. The enhancement of infectivity by NTD mutations suggests that these alterations may facilitate viral entry through mechanisms independent of direct receptor engagement, possibly by modulating spike protein conformation, stability, or interactions with host factors [35]. This finding aligns with recent structural studies indicating that Omicron’s NTD mutations remodel the antigenic landscape of the spike protein, potentially affecting its accessibility and function [36]. The increased infectivity associated with NTD mutations may contribute to the heightened transmissibility of the Omicron BA.1 lineage, providing a selective advantage even in the face of widespread immunity [37, 38]. Structural studies have shown that these mutations not only alter the antigenic surface of the spike but also affect its functional properties, such as receptor binding and membrane fusion [39-41]. For example, Omicron S requires higher levels of ACE2 for efficient membrane fusion [42].

In contrast to the NTD, mutations within the RBD of BA.1 were found to significantly reduce pseudoviral infectivity (Figs. 2 and 3), demonstrating significant trade-off between viral fitness and immune evasion [43]: the combination of all five major escape mutations is “strongly deleterious” to ACE2 affinity. This reduction is likely attributable to alterations in the spike-ACE2 interaction or to conformational changes that compromise the efficiency of viral entry. Notably, the infectivity-enhancing effect of NTD mutations was attenuated when combined with RBD mutations, highlighting a complex interplay between different spike domains (Fig. 2). Despite their deleterious impact on infectivity, RBD mutations, particularly those within the receptor, binding motif (RBM)-were the primary drivers of escape from neutralizing antibodies (Figs. 3 and 4), including therapeutic monoclonal antibodies such as Casirivimab and Imdevimab. Pseudoviruses harboring RBD or RBM mutations exhibited IC_50_ values exceeding the detection limit for these antibodies, indicating robust resistance. This dual effect-reduced infectivity but enhanced immune evasion-suggests that Omicron’s evolutionary trajectory was shaped by intense immune pressure, favoring mutations that allow the virus to circumvent antibody-mediated neutralization even at the cost of entry efficiency [44]. Therefore, the robust resistance of Omicron BA.1 to widely used monoclonal antibodies such as Casirivimab and Imdevimab (Figs. 3 and 4) highlights the need for continuous surveillance of spike mutations and the development of next-generation therapeutics. Antibodies targeting conserved regions outside the highly mutable RBM, or those capable of recognizing multiple spike conformations, may offer broader protection against emerging variants.

The observed trade-off between infectivity and immune escape in Omicron BA.1 highlights a fundamental principle of viral evolution under immune pressure. While mutations that confer resistance to neutralizing antibodies may impair viral entry or replication, the selective advantage conferred by immune evasion can outweigh the fitness costs associated with reduced infectivity-especially in immune-experienced populations. This dynamic is exemplified by the attenuation of infectivity observed with RBD mutations (Fig. 2), which is counterbalanced by the enhanced resistance to antibody neutralization (Figs. 3 and 4). Interestingly, the infectivity-enhancing effect of NTD mutations is diminished when combined with RBD mutations, suggesting that compensatory mechanisms may exist to balance the opposing effects of mutations in different spike domains. This interplay may help explain the complex mutational landscape of Omicron BA.1 and its ability to maintain sufficient transmissibility while evading immune responses. Recent studies have shown that Omicron BA.1 is less virulent than previous variants, a phenotype attributed not only to spike protein mutations but also to changes in non-spike proteins such as nsp6 [45, 46]. While the spike protein is the main driver of immune escape, attenuation of pathogenicity appears to require additional mutations outside the spike, underscoring the multifactorial nature of viral fitness and disease severity [47-49].

While our study provides valuable insights into the functional consequences of Omicron BA.1 spike mutations, several limitations should be acknowledged. First, the use of pseudovirus systems, while informative, may not fully recapitulate the complexity of authentic viral infection in vivo. Second, our analysis focused primarily on the spike protein, whereas other viral proteins (*e.g.*, nsp6) also contribute to variant phenotypes such as attenuation and altered pathogenicity. Finally, the interplay between humoral [50] and cellular immunity [51] in shaping variant fitness warrants further investigation [52], particularly in the context of population-level immunity and vaccine-induced protection [53].

In summary, our domain-specific analysis of the Omicron BA.1 spike protein reveals a nuanced interplay between mutations that enhance infectivity (NTD) and those that drive immune escape (RBD), with the latter conferring robust resistance to neutralizing antibodies at the expense of viral entry efficiency. This evolutionary trade-off underpins the epidemiological success of Omicron BA.1, enabling its rapid global spread despite lower intrinsic infectivity. These findings underscore the importance of continuous genomic surveillance, flexible vaccine strategies, and the development of broadly protective therapeutics to address the ongoing challenge of SARS-CoV-2 evolution.

## Figures and Tables

**Table 1:** Mutations found in the BA.1 spike protein.